# Improved *Neisseria gonorrhoeae* culture media without atmospheric CO_2_

**DOI:** 10.1007/s00253-025-13449-7

**Published:** 2025-03-21

**Authors:** Chukwuma Jude Menkiti, Lori A. S. Snyder

**Affiliations:** School of Life Sciences, Pharmacy, and Chemistry, Kingston University, Penrhyn Road, Kingston Upon Thames, KT1 2EE UK

**Keywords:** *Neisseria*, Sodium bicarbonate, CO_2_, GC agar, Chocolate agar, Thayer-Martin agar

## Abstract

**Abstract:**

Bacterial culture on solid media is the crucial step in diagnosing *Neisseria gonorrhoeae* infections and is the gold standard for determining their antimicrobial resistance profile. However, culture of *Neisseria* spp. can be challenging in resource poor areas, relying on specialist incubators or other methods of supplying 5% CO_2_ for growth of the bacteria. Even when such incubators are available, the CO_2_ to run them may be scarce; there were CO_2_ shortages during the COVID-19 pandemic, for example. Although culture jars with gas packs or candles can be used, these are inefficient in terms of use of incubator space and researcher time. To achieve simplicity in culturing of *N. gonorrhoeae,* the standard Oxoid GC agar base medium, made with the Kellogg’s glucose and iron supplements was improved with the addition of 0.75 g/l sodium bicarbonate (NaHCO_3_), which is inexpensive and readily available. This improved media in a standard incubator performed as well as standard Oxoid GC agar media with supplements in a 5% CO_2_ incubator. Chocolate agar and Thayer-Martin agar with sodium bicarbonate were also developed, with all showing good growth of *N. gonorrhoeae* without the need for atmospheric CO_2_.

**Key points:**

*• Neisseria spp. (N. gonorrhoeae, N. meningitidis) require atmospheric CO*_*2*_ *to grow.*

*• Sources of CO*_*2*_ *may be scarce depending on geography and lab supply availability.*

*• We have developed GC, Chocolate, and Thayer-Martin media that does not need CO*_*2*_ *.*

## Introduction

*Neisseria* species are fastidious organisms and therefore require additional nutritional requirements in the form of supplementary iron (1/1000 v/v) and a glucose-based (1/100 v/v/) supplement to be added to the base GC agar media to grow (Kellogg et al. 1963). The optimum temperature for the growth of *Neisseria* spp. is 35 to 37 °C; they grow best at a pH of 7 to 7.5 (Griffin and Rieder 1957; Welton et al 1944). *Neisseria* spp. are carboxyphilic (capnophilic) bacteria; they require an atmosphere composed of oxygen and a raised level of CO_2_ and therefore grow best in an atmosphere enriched with 5–10% CO_2_ (Griffin and Racker 1956; Tuttle and Scherp 1952). This atmosphere can be provided for agar plate culture using a CO_2_ incubator, which adds CO_2_ gas via a regulator, by using a CO_2_ generating GasPak kit, by lighting a candle in a sealed culture jar, or by marble chips which can also be used to provide CO_2_ enriched atmosphere (Griffin and Racker 1956; Griffin and Rieder 1957; Jones and Talley 1977; Talley and Baugh 1975).

Enabling laboratories to grow *N. meningitidis* and *N. gonorrhoeae* without atmospheric CO_2_, as easily as many other pathogens, opens up prospects for wider coverage of diagnostic and antimicrobial susceptibility tests, particularly in resource poor areas. This would mean a significant improvement in patient care and the potential for treatments to be personalised and targeted. This has been achieved by modifying the GC agar medium (Oxoid) with Kellogg’s supplements (Kellogg et al. 1963) using 0.75 g/l sodium bicarbonate (NaHCO_3_), a carbonic acid with alkalinizing and electrolyte replacement properties.

GC agar (Johnston 1945), Chocolate agar (McLeod et al 1927), and Thayer-Martin agar (Thayer and Martin 1964) are used for the cultivation of *N. gonorrhoeae* and other *Neisseria* spp. in the research laboratory and in diagnostics. We modified these three media with NaHCO_3_ and successfully grew *Neisseria* isolates without the addition of atmospheric CO_2_. Ultimately, a growth media for culturing *N. gonorrhoeae* without the need for atmospheric CO_2_ supplementation will be useful for laboratory research and diagnostics worldwide. This is especially important in resource poor areas, where CO_2_ may not be readily available or in short supply.

## Materials and methods

### Bacterial strains

*N. gonorrhoeae* strain NCCP11945 was obtained directly from those who isolated it via a Materials Transfer Agreement. It can be sourced from the authors of the genome sequencing paper (Chung et al. 2008). Commensal isolates are available from the National Collection of Industrial Food and Marine Bacteria (NCIMB, Aberdeen): *N. subflava* KU1003-01 as NCIMB 15045 AND 15046; *N. subflava* KU1003-02 as NCIMB 15047; *N. cinerea* RH3002v2f as NCIMB 15043; and *N. subflava* RH3002v2g as NCIMB 15044).

### GC agar medium

The GC agar medium (Johnston 1945), prepared according to Oxoid manufacturer’s instructions and supplemented with Kellogg’s glucose and 5% iron supplements (Kellogg et al 1963), was used for the culture of all *Neisseria* spp. used in these investigations. Briefly, 9 g of Oxoid GC Agar Base (CM0367; made of special peptone 15 g, corn starch 1 g, sodium chloride 5 g, dipotassium hydrogen phosphate 4 g, potassium dihydrogen phosphate 1 g, and agar 10 g; pH 7.2 ± 0.2 at 25 °C) was added to 250 ml distilled water and sterilised by autoclaving at 121 °C for 15 min. When the media was cool, but not solidified, 2.5 ml of Kellogg’s glucose supplement (dissolve 40 g D-glucose in 70 ml dH_2_O on a warm plate until dissolved, cool, add 1 g L-glutamine and 2 mg thiamine pyrophosphate (co-carboxylase, bring volume to 100 ml, filter sterilise, and store at 4 °C) and 250 μl of Kellogg’s iron supplement (0.05 g Fe(NO_3_)_3_ in 10 ml dH_2_O, filter sterilised, stored at 4 °C) were added. Media as immediately poured into sterile 90-mm petri dishes and stored at 4 °C once solidified. For the modified GC agar medium, 0.75 g/l of sodium bicarbonate (NaHCO_3_) was added after autoclaved GC media had cooled before pouring into petri dishes, at the same time as adding the glucose and iron supplements. All agar plates were stored at 4° and allowed to return to room temperature before use.

### Chocolate agar medium

Chocolate agar (McLeod et al. 1927) was made using Oxoid GC agar base according to manufacturer’s instructions, including 2% haemoglobin supplement (Thermo Scientific™ Oxoid™ Hemoglobin Soluble Powder). After autoclaving and combining, BD BBL™ IsoVitaleX™ Enrichment supplement was added to the combined GC agar base/haemoglobin. Also added were 15 g/l Tryptic Soy Broth (BD Bacto™), 1 g/l 1-Allyl-3-methylimidazolium chloride (Alfa Aesar™), 5 g/l Sodium Chloride (Fisher BioReagents), 4 g/l Potassium Phosphate Dibasic (Fisher BioReagents), and 1 g/l Potassium Phosphate Monobasic (Fisher BioReagents). For the modified Chocolate agar medium, 0.75 g/l of sodium bicarbonate was added along with the other chocolate agar supplements. All agar plates were stored at 4 °C.

### Thayer-Martin agar medium

For Thayer-Martin agar media (Thayer and Martin 1964), GC agar base (Oxoid) and 2% of haemoglobin were prepared and mixed together as for Chocolate agar. Added to this was IsoVitaleX™ Enrichment (BD BBL™), 3 µg/l Vancomycin Supplement (Oxoid), 7.5 µg/l Colistin sulphate salt (ACROS Organics™), 12.5 µg/l Nystatin (MP Biomedicals™), and 5.0 µg/l Trimethoprim lactate (Alfa Aesar). For the modified Thayer-Martin agar medium, 0.75 g/l of sodium bicarbonate was added along with the other supplements. After cooling and solidifying, all plates were stored at 4 °C.

### *Neisseria* spp. isolates

Five isolates were used for this study: *N. gonorrhoeae* strain NCCP11945 (Chung et al 2008); *Neisseria subflava* isolates KU1003-01, KU1003-02, and RH3002v2g; and *Neisseria cinerea* isolate RH3002v2f (Calder et al 2020). Petri dishes were streaked with 0.5 McFarland solutions calibrated isolates. Identification tests were done throughout the experiments, to verify the *Neisseria* spp. cultures had not become contaminated, including verification of colonial morphology, Gram staining (Smith and Hussey 2005), catalase (Reiner 2010), and oxidase testing (Shields and Cathcart 2010). All of the *Neisseria* spp. investigated here appeared as Gram-negative diplococci under microscopy and are oxidase and catalase positive.

### Colony count

The semiquantitative standard loop method was used to characterise the isolate colonial growths (Ochei and Kolhatkar 2000). Briefly, a 10 µl wire loop was used to inoculate and streak media plates. After incubation, the number of colonies were counted and multiplied by the innoculum size (Ochei and Kolhatkar 2000; Table 1).

**Table 1:** Bacterial growth quantification

Growth characterisation	Growth (CFU/ml)
Heavy	≥ 10^5^ (≥ 1000 CFU)
Moderate	10^4^–10^3^ (100–999 CFU)
Light	10^3^–10^2^ (10–99 CFU)

### *Neisseria* spp. culture media investigations

For the study*,* twelve agar plates (6 standard and 6 NaHCO_3_ modified) were used for each isolate for each of GC, Chocolate, and Thayer-Martin medias. To assess any potential and variations in bacterial growth, 3 standard and 3 NaHCO_3_ modified inoculated plates were incubated in 5% CO_2_ incubator. Under this atmospheric condition, it is expected that *Neisseria* spp. will grow (Griffin and Rieder 1957; Kellogg et al 1963). The other 6 plates, 3 standard and 3 NaHCO_3_ modified, were inoculated and placed in a standard incubator, being provided with a normal atmosphere. The use of 3 plates for each condition produced technical replicates. In addition, the experiment was repeated three times on different days, generating biological replicates.

To check the effect of sealing the inoculated media in an airtight plastic bag on the growth of these *Neisseria* spp., NaHCO_3_ modified media were also inoculated with the isolates and incubated in the 5% CO_2_ and standard incubators at 37 °C for 24–48 h while placed in a separate sealed clear (250 mil thick and 7.5 × 7.5 in size) Alpha Packaging™ Polypropylene grip Sample Bag.

Controls were used in all the experiments and were incubated alongside the media being investigated. A standard GC agar plate inoculated with *N. gonorrhoeae* strain NCCP11945 was used as control; this gonococcal strain is known to require CO_2_ for growth on GC media (Chung et al 2008). The same principles and protocols were also applied to Chocolate agar and Thayer-Martin agar and the NaHCO_3_ modified versions of these media.

### Pili

Dissecting microscope Moticam 1080 HDMI & USB which uses LED light and fibre optical was used to observe for the presence or absence of pili. Single colonies of the isolates on the GC agar media were observed under the dissecting microscope. Colonies with domed, irregular/pointed edges and/or a ring at the edge (signs of piliation) were subcultured onto modified GC agar media after which they were examined under the dissecting microscope for signs of piliation.

## Results

**Fig. 1:**
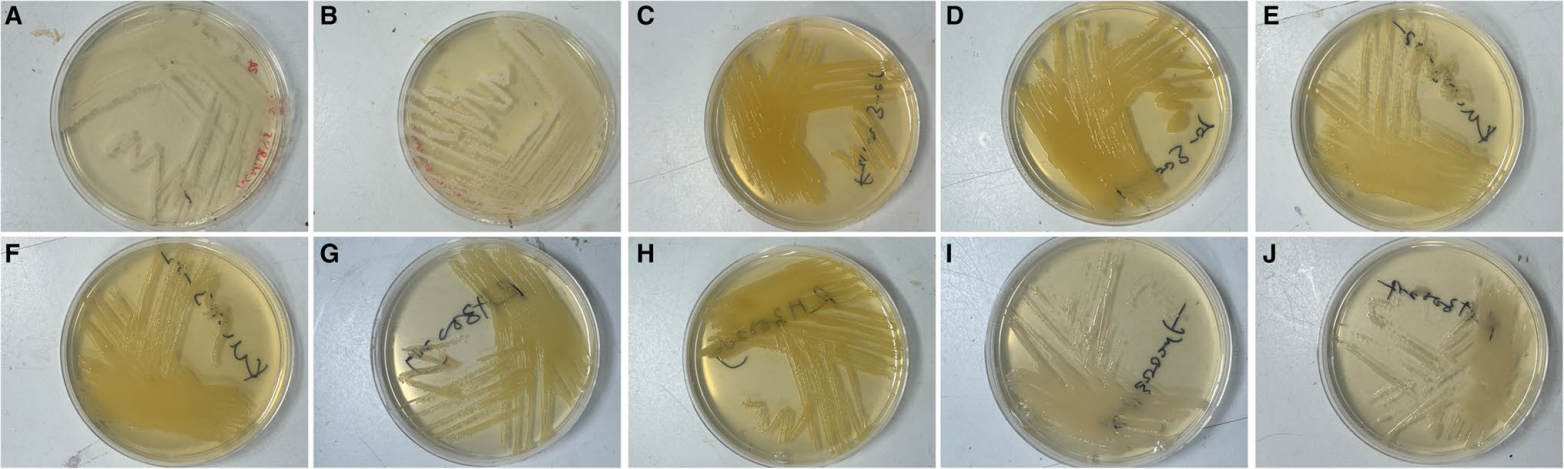
Images of *Neisseria* species isolates grown on GC agar in 5% CO_2_ and on modified GC media without supplementary atmospheric CO_2_. **a** NCCP11945 on GC media. **b** NCCP11945 on modified GC media. **c** KU1003-01 on GC media. **d** KU1003-01 on modified GC media. **e** KU1003-02 on GC media. **f** KU1003-02 on modified GC media. **g** RH3002vg on GC media. **h** RH3002vg on modified GC media. **i** RH3002vf on GC media. **j** RH3002vf on modified GC media

To determine whether the NaHCO_3_ supplemented media were able to support growth as well as standard GC agar media with Kellogg’s supplements, Chocolate agar, and Thayer-Martin agar media, these media and their NaHCO_3_ supplemented versions were incubated in a CO_2_ incubator and the results compared. The *N. gonorrhoeae*, *N. subflava*, and *N. cinerea* grew equally well at 37 °C in all six conditions in the 5% CO_2_ incubator: standard GC agar and NaHCO_3_ supplemented GC agar (Table 2; Fig. 1); Chocolate agar and NaHCO_3_ supplemented Chocolate agar (Table 3); and Thayer-Martin agar and NaHCO_3_ supplemented Thayer-Martin agar (Table 4). In standard atmospheric conditions without supplemental atmospheric CO_2_, there was no growth on GC agar (Table 2), Chocolate agar (Table 3), or Thayer-Martin agar (Table 4). However, the NaHCO_3_ supplemented GC agar, NaHCO_3_ supplemented Chocolate agar, and NaHCO_3_ supplemented Thayer-Martin agar did support growth under standard atmospheric conditions (Tables 2, 3, and 4). The experiments were repeated using three plates per experiment and repeated three times on different days for reproducibility.

**Table 2:** *Neisseria* spp. growth on NaHCO_3_ modified GC agar

	Growth in CO_2_ incubator^b^		Growth in standard incubator^b^	
*N. gonorrhoeae*	24 h	48 h	24 h	48 h
GC agar	+ + +	+ + +	−	−
NaHCO_3_ modified GC agar	+ + +	+ + +	+ + +	+ + +
*N. subflava*^a^	24 h	48 h	24 h	48 h
GC agar	+ + +	+ + +	−	−
NaHCO_3_ modified GC agar	+ + +	+ + +	+ + +	+ + +
*N. cinerea*	24 h	48 h	24 h	48 h
GC agar	+ + +	+ + +	−	−
NaHCO_3_ modified GC agar	+ + +	+ + +	+ + +	+ + +

^a^Data was the same for all three strains of *N. subflava* investigated

^b^Growth: − , no growth; + , light growth; +  + , moderate growth; +  +  + , heavy growth

Data represents triplicate technical and triplicate biological replicates

**Table 3:** *Neisseria* spp. growth on NaHCO_3_ modified Chocolate agar

	Growth in CO_2_ incubator^b^		Growth in standard incubator^b^	
*N. gonorrhoeae*	24 h	48 h	24 h	48 h
Chocolate agar	+ + +	+ + +	−	−
NaHCO_3_ modified Chocolate agar	+ + +	+ + +	+ +	+ + +
Modified Chocolate, sealed bag	+ + +	+ + +	+ + +	+ + +
*N. subflava*^a^	24 h	48 h	24 h	48 h
Chocolate agar	+ + +	+ + +	−	−
NaHCO_3_ modified Chocolate agar	+ + +	+ + +	+ +	+ + +
Modified Chocolate, sealed bag	+ + +	+ + +	+ + +	+ + +
*N. cinerea*	24 h	48 h	24 h	48 h
Chocolate agar	+ + +	+ + +	−	−
NaHCO_3_ modified Chocolate agar	+ + +	+ + +	+ +	+ + +
Modified Chocolate, sealed bag	+ + +	+ + +	+ + +	+ + +

^a^Data was the same for all three strains of *N. subflava* investigated

^b^Growth: − , no growth; + , light growth; +  + , moderate growth; +  +  + , heavy growth

Data represents triplicate technical and triplicate biological replicates

**Table 4:** *Neisseria* spp. growth on NaHCO_3_ modified Thayer-Martin (TM) agar

	Growth in CO_2_ incubator^b^		Growth in standard incubator^b^	
*N. gonorrhoeae*	24 h	48 h	24 h	48 h
Thayer-Martin (TM) agar	+ + +	+ + +	−	−
NaHCO_3_ modified TM agar	+ + +	+ + +	+ +	+ + +
Modified TM, sealed bag	+ + +	+ + +	+ + +	+ + +
*N. subflava*^a^	24 h	48 h	24 h	48 h
Thayer-Martin agar	+ + +	+ + +	−	−
NaHCO_3_ modified TM agar	+ + +	+ + +	+ +	+ + +
Modified TM, sealed bag	+ + +	+ + +	+ + +	+ + +
*N. cinerea*	24 h	48 h	24 h	48 h
Thayer-Martin agar	+ + +	+ + +	−	−
NaHCO_3_ modified TM agar	+ + +	+ + +	+ +	+ + +
Modified TM, sealed bag	+ + +	+ + +	+ + +	+ + +

^a^Data was the same for all three strains of *N. subflava* investigated

^b^Growth: − , no growth; + , light growth; +  + , moderate growth; +  +  + , heavy growth

Data represents triplicate technical and triplicate biological replicates

After 24 h of incubation, there was heavy growth of the *Neisseria* spp. on all the inoculated media incubated in the CO_2_ incubator (Tables 2, 3, and 4). For the media incubated in the standard incubator, there was only growth on the NaHCO3 modified media (Tables 2, 3, and 4). There was moderate growth at 24 h of incubation and heavy growth at 48 h of incubation on the modified Chocolate (Table 3) and modified Thayer-Martin (Table 4) agar plates incubated in the standard atmosphere incubator.

The colonial morphology of the isolates was as expected for each of the *Neisseria* spp. when grown under standard conditions. After growing on the sodium bicarbonate modified agar media, there were no changes to the colonial morphology of the isolates.

**Fig. 2:**
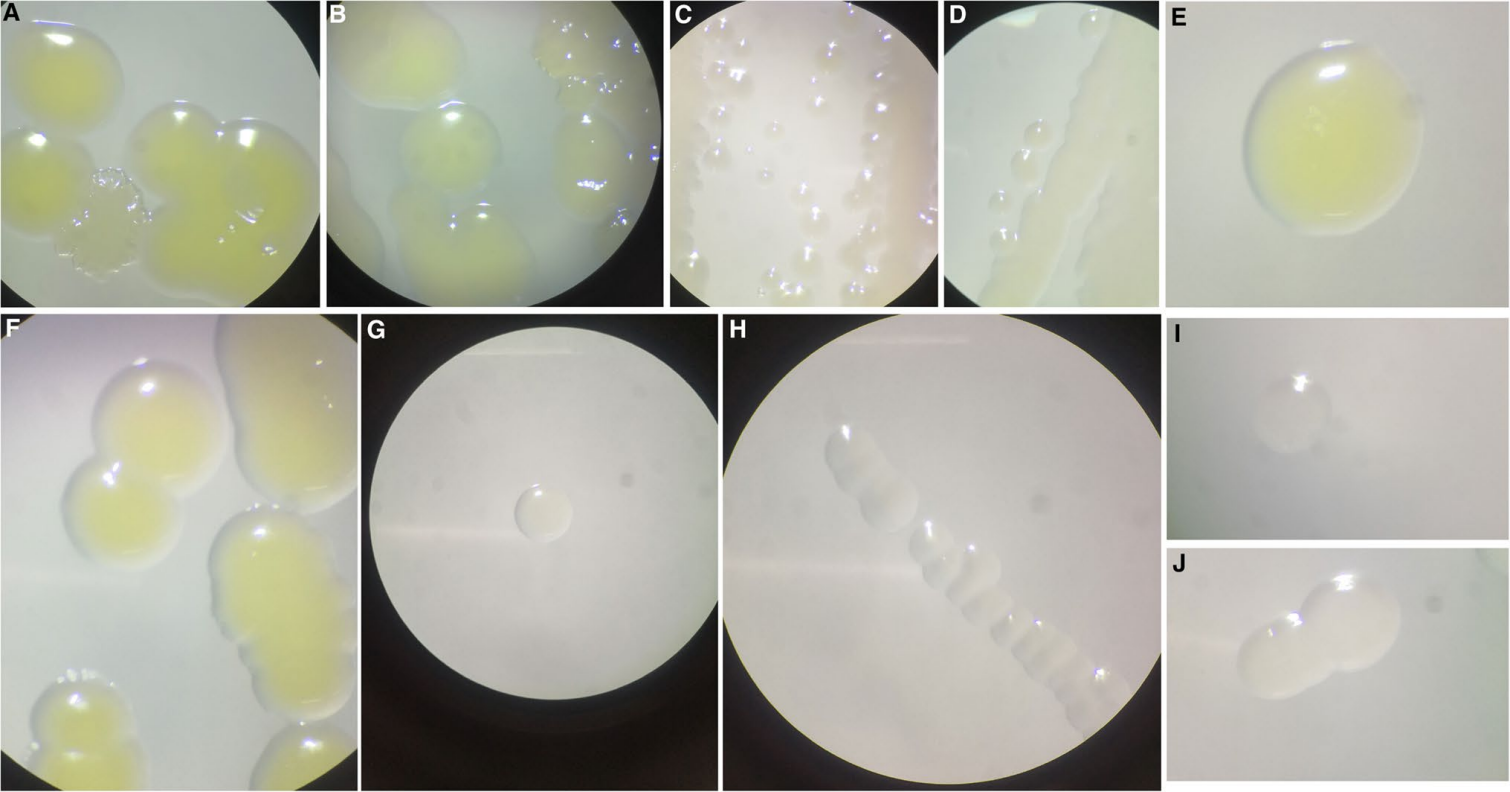
Dissecting microscopy images of *Neisseria* species isolates grown on GC agar with 5% CO_2_ and modified GC media without supplemental atmospheric CO_2_. **a** KU1003-01 on GC media. **b** KU1003-01 on modified GC media. **c** KU1003-02 on GC media. **d** KU1003-02 on modified GC media. **e** RH3002vg on GC media. **f** RH3002vg on modified GC media. **g** RH3002vf on GC media. **h** RH3002vf on modified GC media. **i** NCCP11945 on GC media. **j** NCCP11945 on modified GC media

To demonstrate that pili possessing colonies can be grown with sodium bicarbonate modified media, the colonies grown on modified media were observed using a dissecting mocroscope. Dissecting microscopy of the *Neisseria* spp. isolates grown on regular (non-modified) GC media showed piliated and non-piliated colonies. Non-piliated colonies were flat and round, with smooth edges and no ring at the edge. The colonies appear large unlike piliated colonies that appear smaller and domed, with irregular/pointed edges and a ring at the edge, as previously described (Hu et al 2020). Piliated colonies were selectively inoculated on modified GC media. After 24-h incubation, the colonies were observed under the dissecting microscope, showing that the colonies appeared piliated (Fig. 2).

## Discussion

In our quest to develop an alternative *N. gonorrhoeae* diagnostic test for laboratories, especially those in resource poor areas, bacterial agar media culture is necessary so that antimicrobial susceptibilities can be determined and patient treatment can be personalised and targeted. Although molecular testing can indicate that a bacterial species is present and advances may be able to accurately determine if antimicrobial resistance genes are present, only a bacterial culture is the current gold standard to verify viable infectious agents and determine expression of antimicrobial resistance.

A crucial step in achieving a wider capacity for *N. gonorrhoeae* culturing in poor areas is finding an alternative to the 5% CO_2_ needed for the incubation and growth of the bacteria (Platt 1976). Wherry and Oliver (1916) were the first to highlight the use and importance of providing partial CO_2_ environment for *N. gonorrhoeae* growth. Other means to provide enhanced CO_2_ has included use of Gaspak (Devaux et al. 1987), use of candle jar (introduced by Chapin (1918) and confirmed by Spink and Keefer (1947)), and use of plastic Bio-bags (Carlson et al. 1980).

In this study, we provided the atmospheric requirement need for gonococcal growth by modifying the GC agar medium (Oxoid) with Kellogg’s supplements (Kellogg et al. 1963) using 0.75 g/l sodium bicarbonate (NaHCO_3_), a carbonic acid with alkalinizing and electrolyte replacement properties. We also modified Chocolate agar and Thayer-Martin agar using 0.75 g/l sodium bicarbonate (NaHCO_3_). *Neisseria* spp. are often grown in diagnostic laboratories on Chocolate agar and Thayer-Martin agar; therefore, it was desirable to develop a modification that would enable culture on these media without CO_2_ as well.

In our research, there was no growth of the *Neisseria* spp. isolates on the standard, unmodified GC (Table 2), Chocolate (Table 3), and Thayer-Martin (Table 4) agar media incubated in the standard atmosphere incubator. All of the *Neisseria* spp. isolates were able to grow heavily on these media in the 5% CO_2_ incubator (Tables 2, 3, and 4). All isolates also grew heavily on the NaHCO_3_ modified GC, Chocolate, and Thayer-Martin agar media in the standard atmosphere incubator and on both types of media in the CO_2_ incubator (Tables 2, 3, and 4).

Rubin et al. hypothesised that the CO_2_ requirement for *Neisseria* spp. growth is dependent on β-carbonic anhydrase (CanB) single substitution (Rubin et al 2023). Carbonic anhydrases are metallo-enzymes that have many functions including accelerating the interconversion of HCO_3_, CO_2_, and H_2_O (Rubin et al 2023). The variant CanB^19E^ is needed for growth in the absence of CO_2_ and has been found in non-pathogenic *Neisseria* species and some* N. gonorrhoeae* (Rubin et al 2023). Although this variant is found in some *Neisseria* spp., due to the population-wide CO_2_ requirement, supplementary CO_2_ is needed when isolating these species, especially in a clinical setting.

Previous studies on the effects of bicarbonate (0.009 M) on growth of *Neisseria gonorrhoeae* in liquid and semisolid media (Chapin 1918; Devaux et al 1987; Martin et al 1974) obtained heavy growth with the modified liquid and semisolid media but the concentration of the NaHCO_3_ used is dependent on the container size (the larger the container, the higher the NaHCO_3_ concentration needed). The solid media used in our research has a standard volume (20 ml), the standard size of the sterile petri dish (90 mm), and standard growth obtained from NaHCO_3_ concentration (0.75 g/l) used in our research; hence, there is no need to change concentration using our method.

Heavier growths were achieved at 24 h by sealing the modified agar plates in a plastic bag. The plastic bag prevents the CO_2_ generated by metabolism of the sodium bicarbonate from being lost to the atmosphere. Excellent growth of *N. gonorrheae* was also obtained from broth cultures sealed in separate tightly sealed plastic bags in the experiment by Talley and Baugh (1975), media cultures sealed in plastic bag experiments by Martin et al. (1974), the NaHCO_3_ media enhancement of the growth *N. gonorrhoea* in a closed environment (sealed jar) experiment by Jones and Talley (1977), and in the evaluation of four methods for isolation of *Neisseria gonorrhoeae* by Carlson et al. (1980). The plastic bag also provides increased humidity within the bag which catalyses CO_2_ generation (Martin et al 1974; Spink and Keefer 1947). This shows that the greater the NaHCO_3_ concentration, the higher the growth of the isolates.

These results demonstrate that the addition of sodium bicarbonate to GC agar, Chocolate agar, and Thayer-Martin agar provides the supplementary CO_2_ required for the growth of *Neisseria* spp. Several other studies across bacterial species have also shown that bicarbonate is the reactive species in the fixation of CO_2_. These studies also show that CO_2_ is actively utilised by biotin enzymes like phosphoenolpyruvate carboxykinase, carboxytransphosphorylase, and pyruvate carboxylase (Carlson et al 1980; Cooper et al 1968; Devaux et al 1987; Higa et al 1976; Teraoka et al 1970; Theodore and Englesberg 1964; Tuttle and Scherp 1952).

**Fig. 3:**
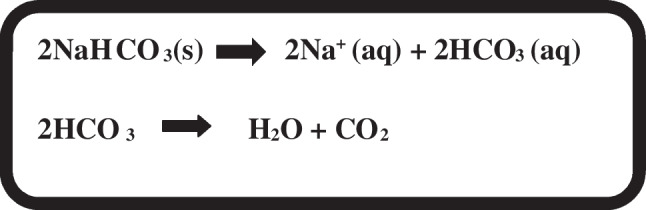
Sodium bicarbonate (NaHCO_3_), in the presence of water decomposes into sodium ions (Na^+^) and carbonate ions (HCO_3_.^−^). Once the carbonate ions are formed, carbon dioxide (CO_2_) is spontaneously released. (s) Soluble compound. (aq) aqueous (dissolved in water)

Hence, sodium bicarbonate-modified media is an alternative to the use of CO_2_ incubators, candle jars, or CO_2_ GasPaks when culturing *N. gonorrhoeae* and other *Neisseria* species. Sodium bicarbonate (NaHCO_3_), commonly known as baking soda, is a monosodium salt of carbonic acid with alkalinizing and electrolyte replacement properties. NaHCO_3_ is water soluble, with two sodium bicarbonates dissociating to form two sodium ions (2Na^+^) and two carbonate ions (2HCO_3_^−^). These carbonate ions decompose to release CO_2_ and H_2_O (Fig. 3). Due to the properties of the media investigated here, the growth conditions are buffered to pH 7.2. The pKa of carbonic acid (H_2_CO_3_) is around 6.4; therefore, the sodium bicarbonate will be in equilibrium within the media, readily converting into CO_2_ during culture. Because the media is buffered and a small amount of sodium bicarbonate is added (0.75 g/l), the pH of the media does not shift from the pH of 7–7.5 required for *N. gonorrhoeae* growth.

This modification of these culture media (supported by the experiments from Talley and Baugh (1975), Jones and Talley (1977)) has shown many advantages over the traditional CO_2_ atmosphere provided by CO_2_ incubator and candle jar by providing excellent growth in 24-h incubation, requiring less incubation space, the NaHCO_3_ concentration can easily be regulated, and the media plates can easily be examined without disrupting the CO_2_ atmosphere of other incubating media plates. With the plastic bag, it can also be used as an alternate transport system. This advance in neisserial bacterial solid growth media alternatives opens up the prospect for more widespread research and diagnostics.

Colony morphology was unchanged between the various media types investigated. Further work will be done to see if the modification has any effect on the genetic make-up or gene regulation of the isolates and also to see if the modified media can be used to grow other bacteria that require the same atmospheric conditions.

Type IV pili, a proteinaceous hair-like appendage expressed by some pathogenic gonococci is a virulence factor involved in gonococcal pathogenesis traits such as motility, DNA uptake during transformation, autoagglutination, colonisation, and host tissue adherence (Swanson et al 1987; Tønjum and Koomey 1997; Hu et al 2020). Pili are also associated with bacterial-bacterial interaction and the formation and dissolution of microcolonies and biofilms (Hu et al 2020).

Morphologically piliated colonies were successfully grown on the sodium bicarbonate modified media. This shows that the modified media supports piliated colonies and the media can be used for transformation of the *Neisseria* isolates. It also shows that the modified media can be used for experiments/works that involves putting bacteria on cells or in infection models.

In the absence of enhanced levels of CO_2_, normally required for laboratory growth of *N. gonorrhoeae*, *N. subflava*, *N. cinerea*, and other *Neisseria* spp., addition of 0.75 g/l to GC media, Chocolate agar, and Thayer-Martin agar is able to support culturing of the *Neisseria* species. The modified media also allows the expression of pili making it suitable for research work. This advance in Neisserial bacterial growth media alternatives opens up the prospect for more widespread research and diagnostics by eliminating the need for costly CO_2_ incubators and gas cylinders that run out at inconvenient times.

## Data Availability

All data supporting the findings of this study are available within the paper.
